# Machine-learning in optimization of CRISPR technology

**DOI:** 10.1088/3049-477X/aea331

**Published:** 2026-09-17

**Authors:** Risi Liyanage, Lei Jin, Shi-Jie Chen

**Affiliations:** Department of Physics and Astronomy, Department of Biochemistry, Institute of Data Science and Informatics, University of Missouri, Columbia, MO, United States of America

**Keywords:** CRISPR technology, machine-learning, artificial intelligence

## Abstract

The clustered regularly interspaced short palindromic repeats (CRISPR)-Cas system has become an indispensable tool in modern gene-engineering applications over recent years. Despite its rapid adoption, further development and broader applicability are hindered by several inherent limitations. This review surveys a range of machine-learning based approaches that aim to address these challenges. In particular, we focus on the optimization of key components of the CRISPR system, including protospacer adjacent motif recognition, Cas-protein-engineering, guide RNA sequence design and extension of the approaches to alternative editing modalities. Applied machine-learning methodologies, their underlying rationale, and comparisons with experimental observations are critically discussed. Special emphasis is placed on the role of machine-learning frameworks in advancing biophysical research, where complex, high-dimensional data increasingly demand integrative computational approaches. Finally, we outline current limitations, draw overarching conclusions, and propose perspectives for future developments and applications in CRISPR-based technologies.

## 1. Introduction

Since its introduction in the early 21st century, [1] the clustered regularly interspaced short palindromic repeats (CRISPR) system has become the cornerstone of targeted genetic-engineering applications. The novelty of this system lies in a paradigm shift from encoding DNA sequence specificity in engineered proteins, such as zinc-finger nucleases [2, 3] and transcription activator-like effector nucleases (TALENs) [4, 5], to encoding target specificity in a programmable single-guided RNA (sgRNA) molecule. This transition represents a fundamental advance in genome-engineering, as RNA-guided targeting circumvents the complex and time-consuming protein-engineering required by earlier approaches [6], with faster synthesis (≈20-nucleotide sgRNAs replacing large protein molecules) and simpler binding rules (ideally, canonical base-pairing rules).

Building upon this programmable targeting framework, subsequent generations of CRISPR technologies have extended its functionality to RNA cleavage (Cas13), transcriptional regulation through CRISPR activation and interference (CRISPRa/i), and precise sequence rewriting via base editing and prime editing systems.

**Figure 1.**
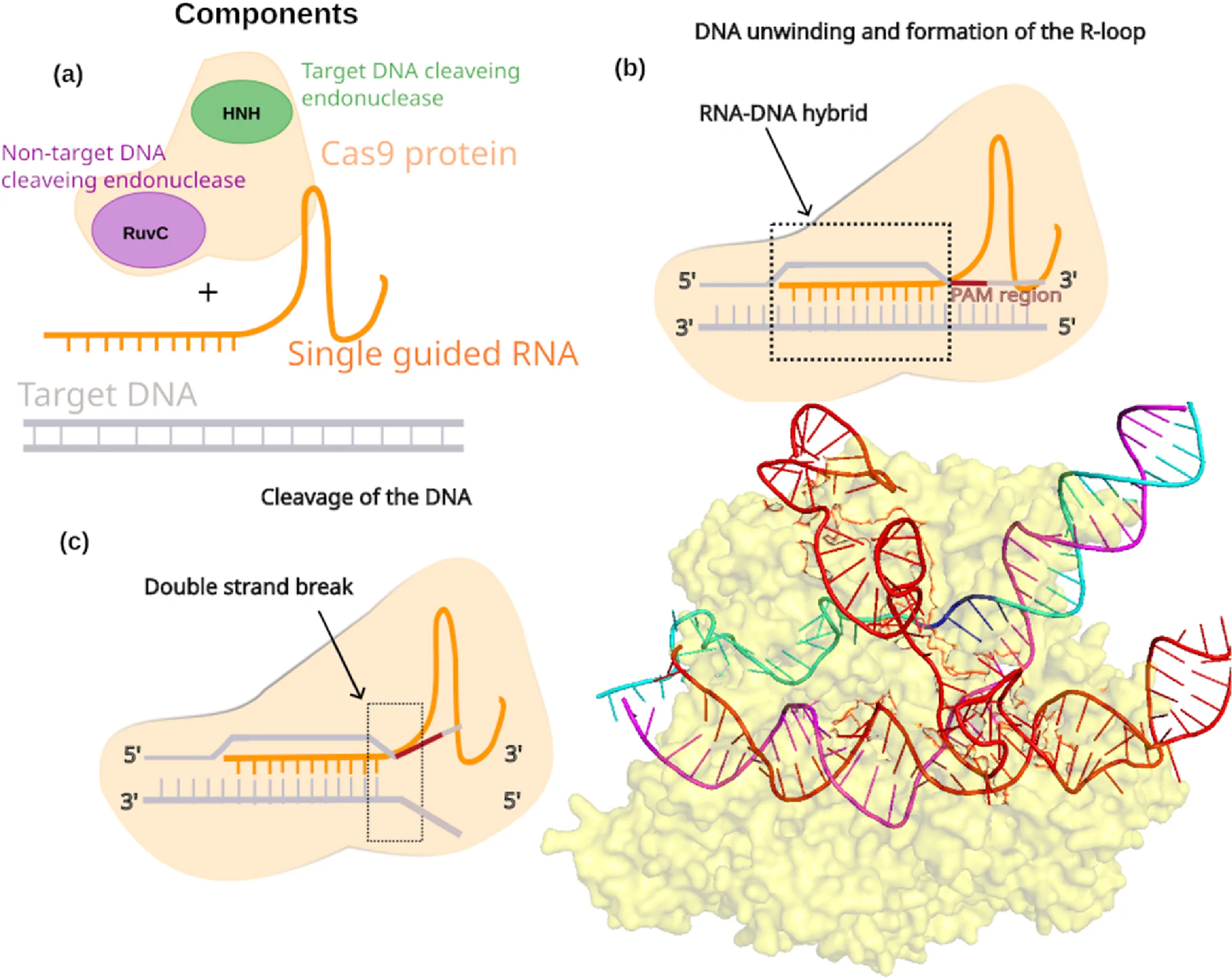
Basic mechanism of the CRISPR-Cas9 system: (a) Major components that create the CRISPR-Cas9 system with principal endonucleases that perform the cleavage. (b) Formation of the R-loop prior to the cleavage action. (c) The occurrence of the double-strand break that is followed by gene knockout or insertion/correction. (d) A color-coded PDB illustration of a fully formed R-loop (PDBID : 5Y36 [29]). The Cas9 protein is shown in green, the target DNA strand in magenta, and the sgRNA in red. The non-target DNA strand is shown in cyan, and the PAM sequence is colored in blue.

The canonical DNA-targeting CRISPR system utilizes a programmable sgRNA to direct a Cas endonuclease (e.g. Cas9 or Cas12) to induce a double-stranded break in the DNA helix (figure 1). Beyond DNA targeting, later CRISPR systems expanded these capabilities to RNA targeting (primarily through Cas13), which led to new applications such as transcript slicing [7]. Furthermore, CRISPR was extended through base and prime editing systems, where cellular toxicity triggered by unintended indels resulting from strand breaks is mitigated through search-and-rewrite operations [8]. Base editing systems differ from canonical systems in that they do not induce strand breaks but instead convert one base to another (C→T or A→G) within a narrow editing window [9, 10]. This is achieved by fusing a catalytically impaired Cas nickase with a deaminase enzyme. Prime editors also employ a Cas nickase but replace the deaminase with a reverse transcriptase and require an extended guide RNA, commonly called a prime editing guide RNA (pegRNA) [11]. This extended pegRNA consists of a primer binding site (PBS) that hybridizes to the nicked target DNA strand to prime reverse transcription. It also contains a reverse transcription template (RTT) containing the desired sequence. This sequence is subsequently copied into the target genome.

Although the CRISPR system has greatly simplified genome-engineering, it is accompanied by several inherent limitations that require careful optimization before real-world and therapeutic applications. One of the primary challenges is off-target activity arising from imperfect sequence specificity, whereby stable non-canonical base pairing between the guide RNA and partially complementary genomic sites can result in unintended DNA cleavage events [12, 13]. Such off-target effects pose a significant concern for therapeutic use. Furthermore, the CRISPR mechanism, particularly that mediated by CRISPR-associated (Cas) nuclease derivatives, relies on the induction of double-strand DNA breaks, which are intrinsically mutagenic [8]. Repair of these breaks requires processing and rejoining of both DNA ends in the absence of an intact template strand, often leading to insertions, deletions, or larger genomic rearrangements. In addition, the requirement for a protospacer adjacent motif (PAM) for target recognition restricts the range of accessible genomic loci, further limiting the flexibility of CRISPR-based interventions [14]. Although the performance of canonical CRISPR systems is largely limited by the aforementioned factors, the emergence of specialized editing platforms such as base editors (BEs) has shifted the optimization landscape. These systems introduce additional factors, including editing-window characteristics, bystander editing, and editor-specific sequence preferences. Meanwhile, diagnostic platforms often depend on collateral cleavage activity and signal amplification [15].

To address this vast space of limitations and optimize CRISPR-based technologies, reliable and systematic approaches for mechanistic investigation are required. While in vivo and in vitro studies remain essential, their high cost, technical complexity, and limited throughput constrain rapid iteration. Consequently, in silico approaches have emerged as a major driving force in advancing CRISPR research [16]. Computational studies of CRISPR systems broadly fall into two categories: biophysical and chemical modeling approaches, such as molecular dynamics simulations [17–20] and kinetic profiling [21], and data-driven methods [22, 23], including machine-learning-based analyses [24–28].

The CRISPR system has benefited substantially from both predictive and generative machine-learning models that target and optimize distinct stages of the CRISPR-Cas workflow. Key determinants of efficient genome editing, including sgRNA binding and cleavage efficiency, off-target activity prediction, Cas protein optimization through sequence mutations, expansion of the PAM space, modeling of structural dynamics and mechanistic behavior, and even base/prime editing applications that mitigate the mutagenic effects of double-strand breaking, have all been addressed using a diverse range of machine-learning approaches.

For a structured perspective on these diverse machine-learning approaches, we organize the review according to the main optimization targets in CRISPR engineering: (i) expansion of the PAM space, (ii) expansion of the Cas effector protein space, and (iii) optimization of guide RNAs. While the broader CRISPR landscape encompasses RNA-targeting and epigenome-editing applications, the majority of currently available machine-learning models have been developed primarily for canonical DNA-targeting CRISPR platforms. Consequently, the major focus of this review remains on DNA-targeting systems, while emerging machine-learning approaches for base and prime editing are discussed separately to emphasize their distinct optimization objectives and methodologies.

Section 2 of the review focuses on expanding the PAM space of CRISPR systems and, consequently, expanding the targetable loci of the genome, specifically across type I, II, and V CRISPR systems. Here, we emphasize methods for both identifying wild-type PAM sequences and probing the potential of generative models to predict probable PAM sequences. Methods for mining naturally occurring Cas effector proteins, as well as generating viable novel effectors, are discussed in section 3. This optimization strategy is more generalized and is relevant across all CRISPR families. We discuss how the conventional classification task of mining metagenomic cassettes has evolved toward deep generative modeling approaches based on large language models (LLMs). Section 4 focuses on cleavage kinetics and cleavage-related modalities (efficiency and specificity) and is targeted primarily toward SpCas9 data-driven models due to the abundance of high-quality data compared with other CRISPR families. It evaluates regression and multi-task deep-learning architectures optimized for a critical translational objective: maximizing on-target cleavage while minimizing off-target genomic toxicity. Finally, section 5 discusses machine-learning approaches for base and prime editing, where optimization extends beyond canonical cleavage activity to editor-specific determinants such as editing windows, bystander editing, editing outcomes, and design strategies. Across these sections, we compare conventional machine-learning approaches, deep-learning architectures, and emerging protein and genomic language models, highlighting how different computational paradigms address distinct biological constraints throughout the CRISPR engineering workflow.

## 2. Expansion of the PAM space leads to a more generalized applicability of the method

Constraints imposed by the requirement for PAM adjacency to the target genomic locus lead to a major reduction in the targetable loci, particularly in DNA-targeting CRISPR systems such as the Cas9 and Cas12 nuclease families. However, due to their architectural simplicity, these class 2 Cas proteins dominate genome-engineering applications. Hence, optimization of the PAM space is considered an important aspect of computational optimization for CRISPR systems, even though it is not universally applicable.

### 2.1. Language models aid in alternate PAM predictions

To address the limitation of PAM recognition, computational approaches that predict probable PAM sequences for a given Cas protein are desirable for expanding the editable genomic loci space and enabling the rational discovery and engineering of novel CRISPR systems.

**Figure 2.**
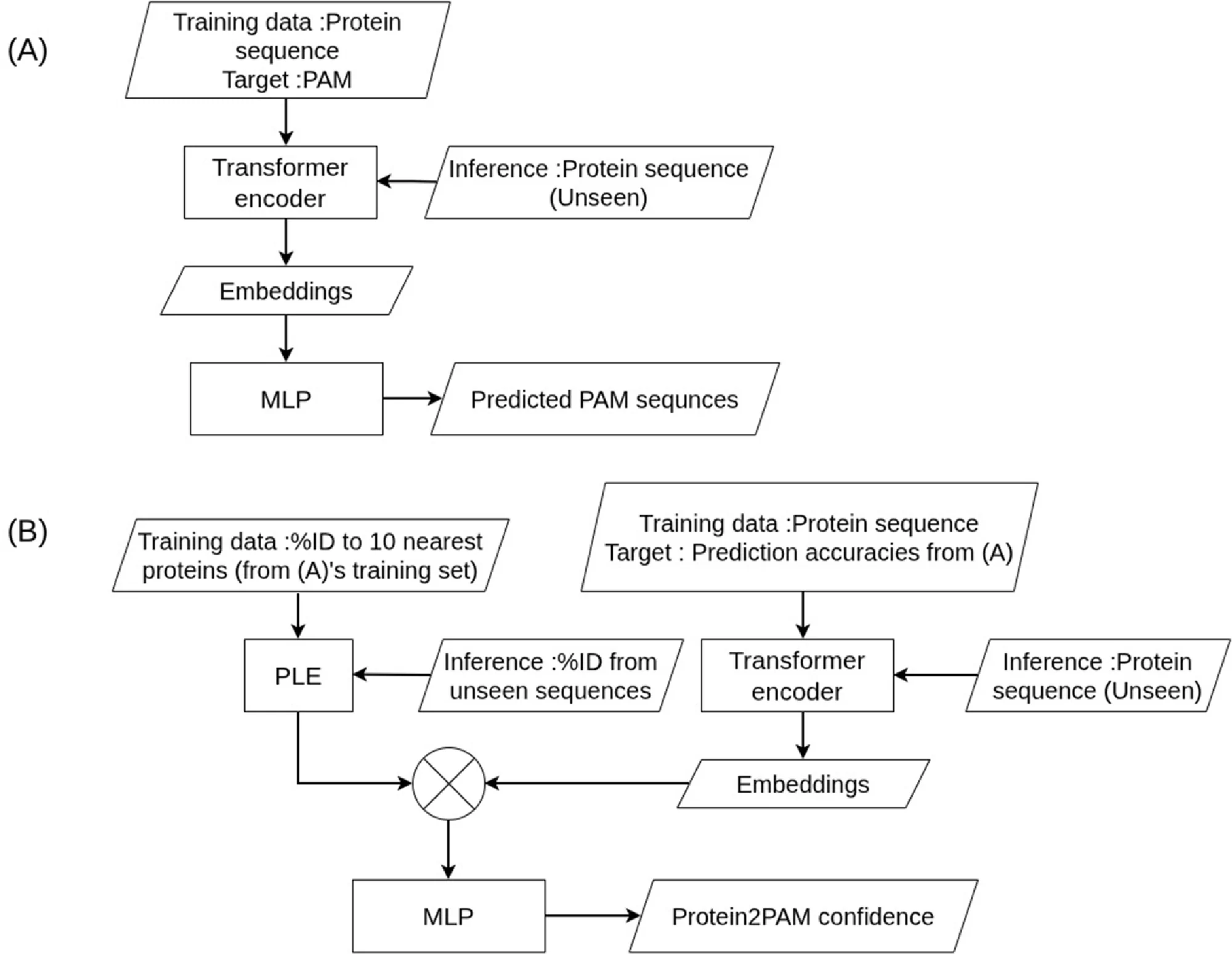
Structural and methodological differences in the two models in Protein2PAM : (A) The primary model-Protein2PAM predicts PAM nucleotide preferences from protein sequences using a transformer encoder and MLP. (B) A secondary model estimates the reliability of each PAM prediction using the primary model’s accuracy data and piecewise linear embeddings (PLE) of the sequence similarities. A combination of the encoded sequence embeddings and PLEs is then fed through the MLP to predict the confidence estimations.

One such model, Protein2PAM [30] utilizes a large dataset of CRISPR-Cas proteins (Type I, II, V—DNA targeting) and corresponding PAM sequences [31] to train a machine-learning model. This dataset substantially expands coverage of PAM-compatible genomic contexts, which are quantified in 10 bp windows. This represents a major improvement in practical targetability, especially since the majority of known Protospacer motifs span a 3–6 bp interval. Using a pre-trained 650 *M*-parameter transformer as a protein language model (pLM) [32], the model learns amino-acid-sequence motifs, co-evolutionary relationships, and functional residues and converts them into high-level embeddings. These embeddings are subsequently mapped to PAM targets using a multilayer perceptron (MLP) [33], which outputs a probability matrix predicting probable PAM sequences (figure 2(A)).

To address uncertainty and assess the reliability of PAM prediction, a secondary model is trained using the primary PAM prediction model, together with piecewise linear embeddings (PLEs) of sequence similarities in the training set (figure 2(B)). Complementing the primary prediction model, the secondary model enables the identification of PAM predictions that are more likely to generalize beyond the observed diversity of Cas proteins. This combined approach yields high cosine similarity on held-out data, indicating strong predictive performance relative to the nearest-neighbor baseline and the advantages of hidden pattern recognition and higher-dimensional embedding in machine-learning-based approaches. Furthermore, validation on experimentally characterized Cas proteins demonstrated strong generalization, including accurate recovery of consensus PAMs.

### 2.2. Machine learning guided PAM space exploration

Another protein sequence to PAM mapping application using machine-learning is demonstrated in the PAM machine-learning algorithm (PAMmla) model [34]. In this study, the sequence space consists of a theoretical library of 64 million variants ($20^6$). The library is generated through saturation mutagenesis of six residues within the SpCas9 PAM-interacting (PI) domain–residues specifically chosen because they modulate specificity at the third and fourth PAM positions. Additionally, the model includes 16 NGNN PAMs ($4^2$), which are created by mutating the same third and fourth positions of the canonical NGGN PAM. Bacterial-based positive selection assays identify Cas9 variants that remain functional under cellular constraints. However, they are limited in scope and can only experimentally characterize a tiny fraction of the accessible sequence space. In contrast, HT-PAMDA [35] is an in vitro assay that can quantitatively measure PAM-dependent cleavage kinetics independently of cellular selection pressures. HT-PAMDA reveals that a substantial proportion of randomly sampled variants retained activity on at least one PAM. This striking disparity highlights the existence of a vast, experimentally inaccessible functional landscape and underscores the need for a machine-learning-based approach.

PAMmla is trained using HT-PAMDA-derived cleavage rate constants across a diverse set of PAM contexts. The model incorporates data from both bacterial selection-derived enzymes and randomly sampled variants to capture functional and non-functional sequence features. The final PAMmla model is a fully connected neural network composed of multiple hidden layers, employing ReLU activation and dropout regularization. This model achieves high predictive accuracy on unseen data, as shown by strong Pearson correlations between predicted and experimentally measured HT-PAMDA rate constants. It also demonstrates robust generalization to engineered SpCas9 variants and protein sequences substantially diverged from the training set. Subsequently, PAMmla is also applied to predict PAM-dependent cleavage profiles for the original 64 million SpCas9 variants, enabling in silico ranking of enzymes by predicted activity or selectivity for each PAM. Experimental validation of top-ranked variants using HT-PAMDA confirms high predictive accuracy and reveals multiple novel PAM classes not recovered by bacterial selection. PAMmla-derived enzymes exhibit substantially higher efficiency and specificity for target PAMs than selection-derived variants. This result demonstrates that machine-learning-guided exploration of sequence space can identify highly tailored Cas9 enzymes beyond those accessible through experimental selection alone.

There exist two fundamental PAM optimization paradigms, namely prediction-based and engineering-based approaches. Prediction-oriented approaches rely on inferring biological relationships between Cas proteins and PAM sequences through representation learning of naturally occurring proteins and subsequent mapping. These approaches (Protein2PAM) employ pLMs and transfer-learning techniques to derive context-aware, high-level embeddings of input sequences. The resulting representations enable the identification of new protein sequences specialized for targeting novel PAMs, thereby expanding the targetable genomic loci space. The use of representation learning mitigates the need for manually engineered descriptors and enables richer sequence representations than traditional feature-engineering approaches. In contrast, PAM-engineering approaches (PAMmla) rely on exploratory and exhaustive behavioral analyses of mutated sequence spaces. These studies suggest that naturally encoded evolutionary information alone may not be sufficient to explore the full breadth of the viable PAM space. They seek to extrapolate mutated Cas enzymes, specifically variants of the PI domain, that can target expanded PAM spaces. Ultimately, both approaches converge on the same machine-learning objective, the extension of the PAM space, while diverging in their underlying hypotheses, which rely on either natural evolution or directed mutagenesis.

## 3. Machine-learning-based approaches for Cas protein identification and enhancement

### 3.1. Searching the annotated genome for protein alternates

Inherent limitations of the readily available Cas proteins such as Cas9, Cas12a, including the generation of double-strand breaks (DSBs), unpredictable indels, large molecular size, and PAM constraints, can restrict applicability and efficiency. These issues motivate the development of alternative proteins that overcome such limitations [36, 37]. Mining for full CRISPR-Cas operons (Cassettes, including Cas proteins, CRISPR array, and tracrRNA) in the annotated genome with the aid of artificial intelligence have therefore become an active area of research. This requires answering two fundamental questions: Where are the cassette boundaries? Which Cas protein types are present within the cassette?

To automate this process, Casboundary [38] uses a machine-learning-based screening procedure. The dataset consists of general and CRISPR-specific hidden Markov model (HMM)- based features [39] and protein properties, with singular value decomposition [40] used for meaningful truncation of the feature set. The algorithm, starting from a signature Cas gene, scans left and right, using an auxiliary classifier to determine whether nearby genes belong to the same CRISPR cassette, and stops when too many consecutive non-Cas genes (>3) are encountered. This Casboundary finding procedure concludes with the left and right limits of a single cassette and utilizes a binary classifier to sort Cas vs non-Cas genes. Both Extremely Randomized Trees (ERT) [41] and deep neural networks (DNN) [42] are employed with grid search hyperparameter tuning for the classification problem.

Contrary to general intuition, for both single- and multi-module cassette-containing genomic loci, ERTs slightly outperform DNNs according to validation metrics such as Jaccard similarity [43] and Cassette Loss (CS), a context-based derivation of mean absolute error. This observation may reflect the fact that boundary identification is a switch-like decision problem at each genomic locus. Accordingly, ERTs may function effectively as rule learners, whereas DNNs are generally better suited to smooth function approximation.

For Cas protein type classification, the same models are again employed, with averaged *F*-score [44] as the evaluation metric. Both models demonstrate high *F*1-scores, with ERT showing slightly better results, and both outperform other HMM- and rules-based models [45]. These results suggest the necessity of machine-learning-based approaches for annotating highly diverged protein families like Cas.

Mining for a specific protein family in meta-genomes is typically performed through sequence alignment, because cassette-level context is often missing or unreliable in fragmented assemblies. However, this approach is highly local and may overlook chimeric or rearranged sequences because it does not incorporate global context, functional plausibility, or residue dependencies. Machine-learning can address this limitation by enabling homolog detection in broader sequence contexts where alignment-based methods may fail.

**Figure 3.**
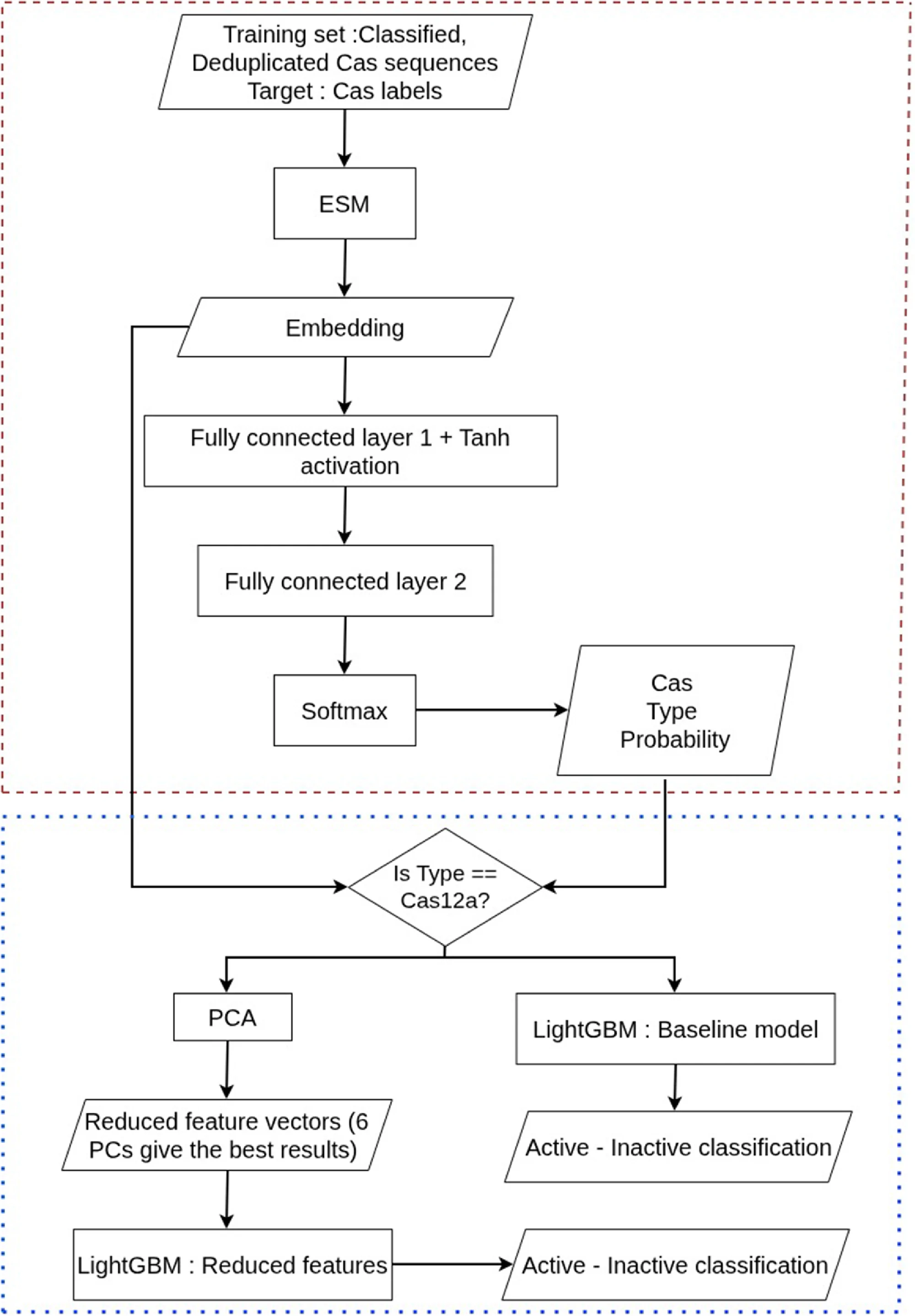
Architecture of AIL-Scan (red) and subsequent trans cleavage activity predictor (blue): ESM is updated with an additional two fully connected layers for the classification problem and a softmax layer for the conversion from logits to probabilities. The trans activity predictor uses Cas12a embeddings from the AIL-Scanned results and trains a LightGBM model to classify active and inactive proteins in the newly identified cases.

Artificial Intelligence-assisted CRISPR-Cas Scan (AIL-Scan) [46] is one such strategy that leverages an Evolutionary Scale language Model (ESM) [47] in mining for CRISPR-Cas-like amino acid sequences in the meta-genomes. A curated dataset of non-redundant, family-wise-classified Cas (positive) and non-Cas (negative) proteins is used in this study to train the ESM for classification. This is done by fine-tuning the ESM-2 model with two added fully connected layers for feature projection and class prediction (figure 3) along with Deepspeed [48] and Accelerate for optimal memory utilization, and FocalLoss to address severe class imbalance. The data and model structure allow the final model to classify not only the corresponding family but also between Cas and non-Cas sequences, making it applicable across genomes and fully automated.

Principal component analysis (PCA) [49] is used to address the classic small-sample, high-dimensional learning problem, and embeddings from the final ESM layers serve as feature vectors for this model. Results indicate that restricting the feature vectors to a compact PCA space of 6–8 dimensions significantly improves accuracy relative to the use of raw embeddings. Among the evaluated models, Light Gradient Boosting Machine (LightGBM) [50] outperforms Categorical Boosting (CatBoost) [51] and random forest (RF). This may be because LightGBM employs leaf-wise growth, which can more efficiently exploit small datasets than level-wise or symmetric growth strategies.

The ESM-based classifier yields highly accurate classification results matching or exceeding HMM-based alignment or hybrid models [52]. Furthermore, the model is applied to predict trans cleavage activity from the Cas12a-like sequence using tree-based learning methods. Despite the sample-size limitations, the model generalizes moderately well to unseen proteins, indicating that ESM embedding outputs have encoded functionally relevant information adequately despite the small training data set.

### 3.2. Artificial intelligence-assisted Cas-protein generation

Among the various limitations of nuclease-based genome editing, the unpredictable insertions and deletions caused by DSBs are particularly problematic, as they can lead to cytotoxicity and genomic instability. While alternative modalities such as base editing address these limitations by avoiding DSB formation, they also provide a platform for engineering improved Cas effectors [9, 10]. The model discussed below focuses on this latter aspect. Instead of predicting editing outcomes, it employs generative machine learning to design novel Cas protein variants that are subsequently incorporated into a base-editing architecture. Thus, although the downstream application is a base-editing system rather than a nuclease-based editor, the learning task is fundamentally one of protein optimization.

OpenCRISPR-1 [31] is a synthetic, high efficiency nuclease designed by harnessing the power of pLMs. Based on concepts from LLMs, pLMs can move beyond explicit evolutionary constructs that constrain classical mutagenesis-based models. In this study, an expanded dataset is assembled, including Cas proteins, trans-activating CRISPR RNAs (tracrRNAs), and PAMs. ProGen2 [53], a pLM trained on over a billion protein sequences, is then fine-tuned in a family-agnostic manner using the CRISPR-Cas atlas to capture CRISPR-specific patterns. The fine-tuned model is subsequently used in an autoregressive loop to generate 2 million unconditional and 2 million family-oriented sequences.

Although the model successfully generates sequences conditional on a family-specific residue pattern, the generated sequences are not necessarily functionally viable, due to the diluted context awareness in fine-tuning procedure. This conclusion is further justified by a two-fold increase in the viability of the generated family-like sequences, when the base pLM is fine-tuned only with a certain family (Cas9), even without conditional prompting. Furthermore, phylogenetic analysis of the generated sequences against natural Cas9 sequences shows that the pLM has captured the full evolutionary grammar of the Cas9 family and has extended the possible Cas9 sequence space while still obeying the viability cutoffs. Notably, one generated sequence was subsequently developed into the base-editing platform OpenCRISPR-1. In contrast to machine-learning models for base editing, which heavily rely on determinant-based system optimization, this model focuses on the generation of viable Cas sequences that extend beyond the naturally observed evolutionary space. Hence, OpenCRISPR-1 represents an example of artificial-intelligence-assisted protein generation.

Protein mining models have effectively simplified the process of identifying feasible Cas proteins from genomic data into a classification problem. Early approaches (Cas-boundary) to Cas protein discovery predominantly relied on hand-engineered sequence features, requiring domain expertise to identify informative biological determinants. Although these approaches were successful, recent advances in machine-learning methods (AIL-scan) have shifted the paradigm toward employing self-supervised models to learn the essential biological patterns and evolutionary information. Furthermore, increasingly used transfer-learning approaches enable the incorporation of Cas-specific context into pre-trained LLMs with minimal fine-tuning. However, for these approaches, training a dense neural network on the limited dataset of available Cas proteins may lead to overfitting [54]. The progression from conventional supervised learning toward LLMs and self-supervised representation learning reflects the shift from task-specific feature extraction to the use of reusable biological representations. Furthermore, recent generative machine-learning models (OpenCRISPR) enable the exploration of previously unseen area of the Cas evolutionary space while adhering to biological constraints. While benefiting immensely from transfer learning, the generative paradigm especially emphasizes the importance of context awareness for the viability of the generated proteins.

## 4. Optimization of gRNA for better binding and cleavage efficiency

Following the target site recognition, guide RNA induced hybridization determines the specificity and stability of the effector recruitment [55, 56]. Given its central role in determining cleavage efficiency and specificity, optimization of gRNA sequence and structure to enhance on-target binding and cleavage activity while suppressing off-target effects has been the subject of extensive research. These in vivo and in vitro studies have generated rich datasets that have enabled the development of numerous machine-learning-based approaches.

### 4.1. Regression-based modeling of on-target activity

One of the earliest and most influential machine-learning models for predicting gRNA on-target cleavage efficiency is Azimuth [57], which employs a boosted regression tree [58] model to predict a continuous sgRNA activity score.

This model is trained on large-scale wild-type CRISPR-Cas9 loss-of-function screening datasets, comprising tens of thousands of gRNAs targeting thousands of genes. In these, the gRNA activity is inferred from normalized depletion measurements reflecting relative on-target cleavage efficiency in cellular viability assays [59]. This study is important because of the framing of guide RNA activity as a regression problem rather than a binary classification task (active vs inactive). The Azimuth framework evaluates multiple machine-learning models, including L1-regularized regression and classification approaches. The enhanced performance of the L1-regularized regression model (Elastic net) over L1-regularized logistic regression (LR)and support vector machine (SVM) [60]-based classification approaches highlights the loss of critical information introduced by arbitrary activity thresholds and the inherent class imbalance in binary labeling schemes. This justifies the usage of a powerful boosted regression trees method in subsequent stages.

### 4.2. Unification of on- and off-target activity for predictive models

While accurate prediction of on-target activity and cleavage efficiency is central to sgRNA optimization, off-target prediction is equally critical for a comprehensive assessment of sgRNA performance.

DeepCRISPR [61] is one such early machine-learning framework that unifies on-target and off-target prediction within a single modeling pipeline. The dataset used for this study contains over 16 000 labeled sgRNAs belonging to four cell types: HCT116, HEK293, HeLa and HL60. Akin to the Protein2PAM model that employs pLM to generate sequence embeddings, DeepCRISPR designs and utilizes a deep convolutional denoising neural network- (DCDNN) based autoencoder [62] to learn sgRNA sequence representations in an unsupervised learning problem. The autoencoder is trained on all NGG PAM–flanked 20-bp DNA sequences across the human genome, enabling the training of encoder weights on rich, context-aware data. These learned representations serve as the foundation for subsequent supervised models.

**Figure 4.**
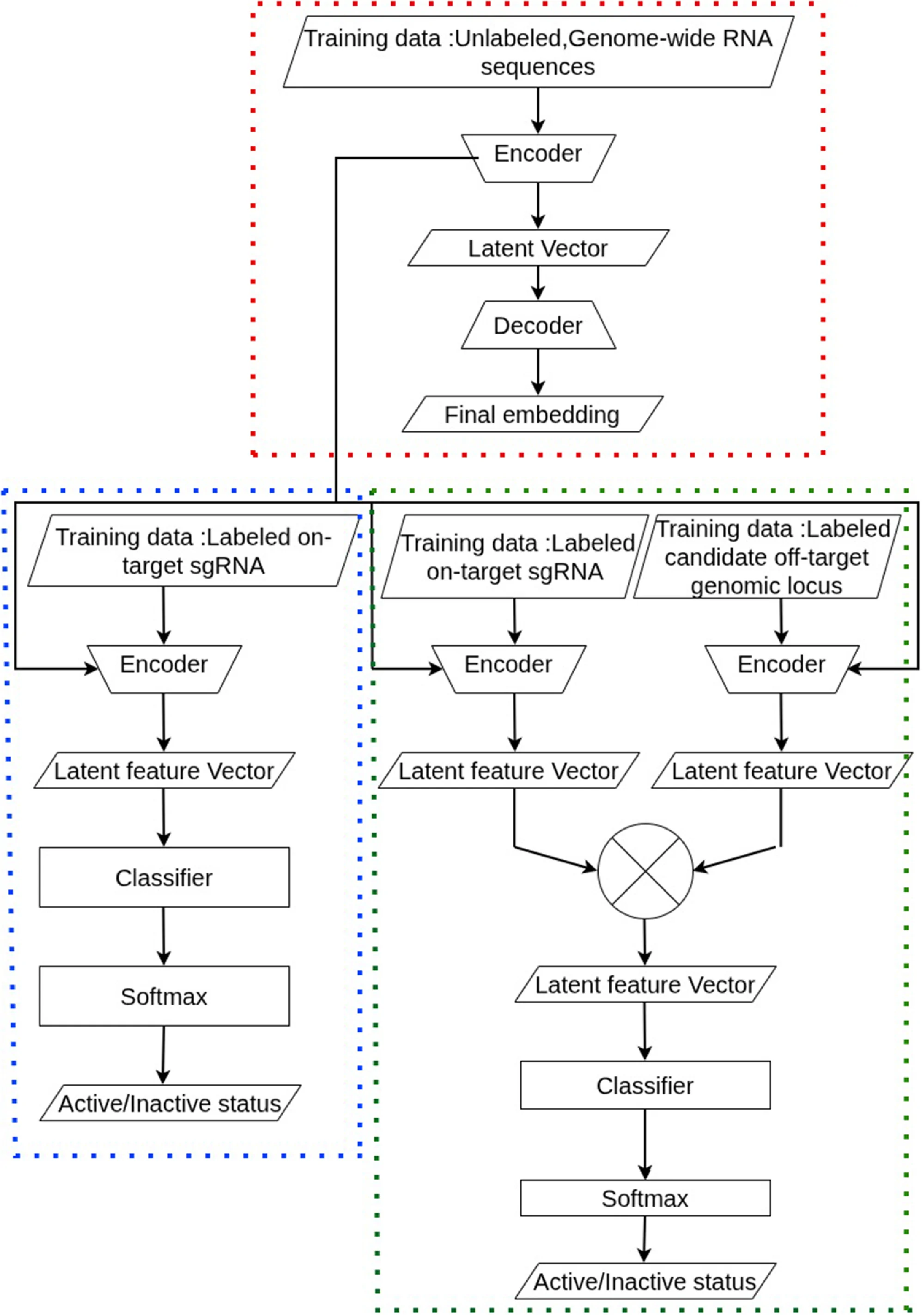
Autoencoder with common encoder head (red), on-target activity predictor (blue) and off-target activity predictor (green) :In the DeepCRISPR framework, first an unsupervised autoencoder is trained, and the encoder is extracted to be used as the embedding head for the on- and off-target convolutional neural network (CNN) classifiers. Encoder weights are fine-tuned during the CNN’s training. The classifier outputs the active or inactive status of the given sgRNA. In the off-target branch, each data point represents two sequences: the sgRNA and the off-target genomic locus, thus an active result corresponding to unintended cleavage in this context.

In DeepCRISPR, the on-target prediction component is implemented as a CNN [63] trained on sgRNAs with experimentally measured cleavage efficiencies, where DCDNN encoder weights are further fine-tuned during supervised training (figure 4). Importantly, authors systematically evaluate multiple architectural variants, including CNN only, DCDNN encoder combined with CNN, and data augmentation combined with DCDNN encoder and CNN, on both regression and classification applications, demonstrating that the full integrated model significantly outperforms other sgRNA activity prediction methods.

Off-target prediction in DeepCRISPR is treated as a pairwise learning problem, where each sample consists of an sgRNA sequence and a candidate off-target genomic locus. Both sequences are independently encoded using a shared pre-trained DCDNN encoder through transfer-learning, and the resulting feature representations are combined and trained through a CNN to predict off-target activity.

The model is trained on approximately 160 000 labeled sgRNA-locus pairs, with bootstrapping integrated into batch training to address severe class imbalance. Both classification and regression formulations are evaluated for a more comprehensive outlook on the off-target assessment problem. Benchmarking analyses show that DeepCRISPR consistently achieves improved predictive performance in terms of receiver operating characteristic—area under the curve (ROC-AUC) [64] across multiple evaluation metrics, indicating the advantages of data-driven machine-learning models over heuristic-based approaches for off-target prediction.

### 4.3. Kinetic mechanism

Unlike end-to-end models designed to predict on- and off-target activities, a recent RF regression model (kCRISPR) reveals the unfolding rate of the R-loop to be a crucial kinetic indicator of cleavage efficiency [26]. The kCRISPR RF model was trained on more than 14 000 sequences spanning 11 on- and off-target datasets. Various kinetic and thermodynamic features were evaluated using the out-of-bag (OOB) error, wherein the datasets were perturbed through random permutations to determine the average change in OOB error for each feature. Through this feature importance analysis, the RF model identified the R-loop unfolding rate as the top-ranked feature and a key predictor of cleavage efficiency.

By predicting both on- and off-target cleavage efficiencies within a unified kinetic framework, the kCRISPR model demonstrates improved predictive performance, particularly for off-target cases. In addition to enhancing accuracy, the model uncovers key mechanistic features of CRISPR-Cas9 targeting and cleavage, such as a two-step kinetic process in R-loop folding/unfolding and a kinetically important intermediate state along the pathway. Ultimately, the kCRISPR model may help pave the way for new strategies in sgRNA optimization.

### 4.4. Feature-engineering for better performance

Feature-engineering is a critical preprocessing step for many machine-learning algorithms. DeepHF [65] utilizes a comprehensive dataset generated from a genome-wide pooled CRISPR screen in human cells, where over 80 000 designed gRNAs targeting protein-coding genes and microRNAs are delivered via lentiviral transduction. On-target activities of these gRNAs for WT-SpCas9, eSpCas9 (1.1) [66], and SpCas9-HF1 [67] are quantified using deep sequencing-based indel measurements following rigorous error filtering and replicate validation.

Using this dataset, the authors systematically extract, evaluate, and rank the importance of 1031 sgRNA features by combining eXtreme Gradient Boosting (XGBoost) with SHapley Additive exPlanations (SHAP) [68]. Feature importance is analyzed in both global and local sequence-specific contexts. This analysis identifies key determinants of gRNA activity, and these insights are further validated through empirical analyses of general sequence and physicochemical properties. Employing a determinant feature set, the study conducts a comprehensive benchmarking of both deep learning and other conventional machine-learning algorithms. Among conventional methods, namely linear regression, L2-regularized regression (ridge) [69], XGBoost and MLP achieve the highest predictive accuracy whereas among deep learning architectures such as CNNs and recurrent neural networks (RNNs) [70], RNN-based models exhibit better performance.

**Figure 5.**
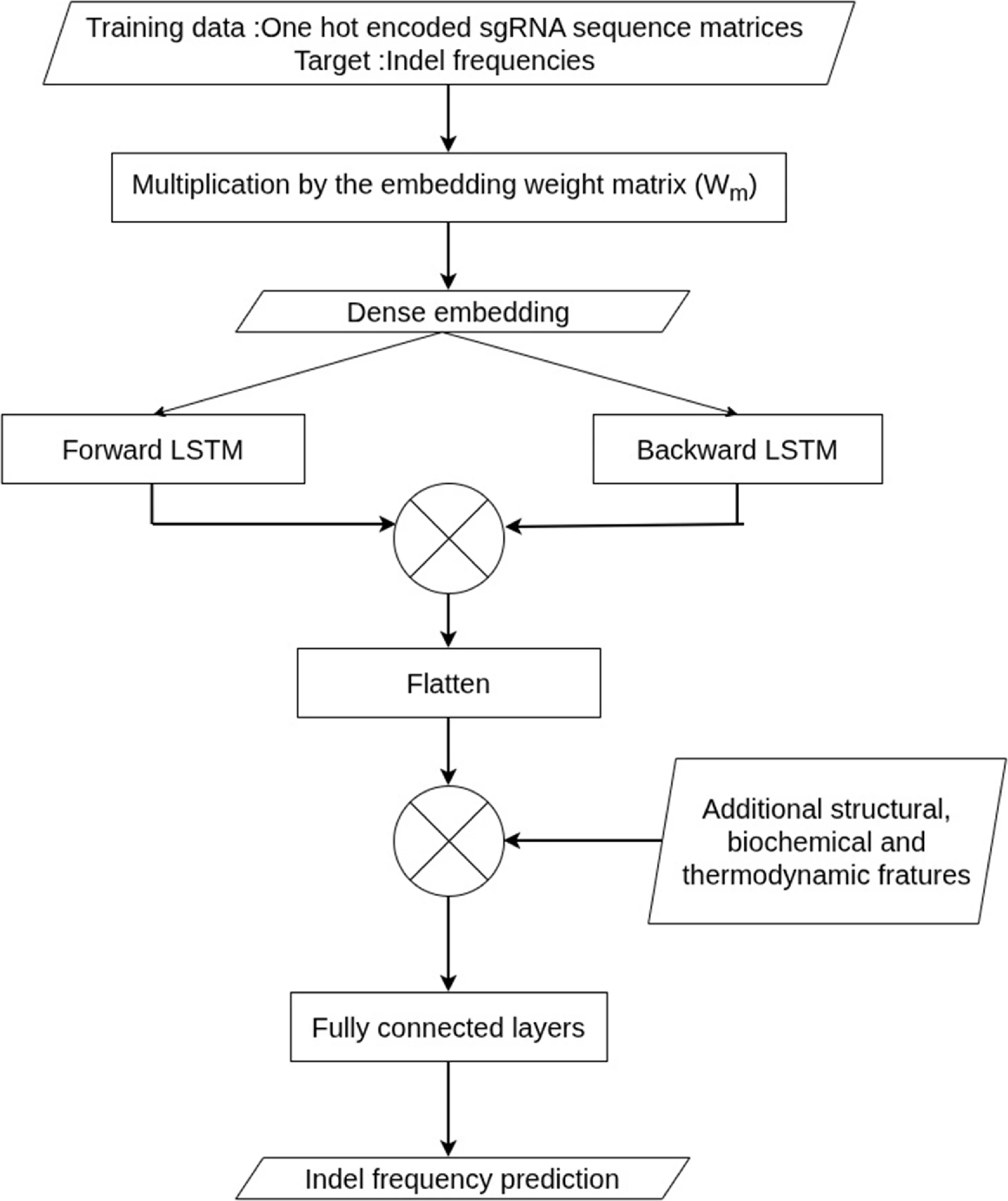
DeepHF main model with BiLSTM RNN layers that capture directional hidden states : The input data for the BiLSTM are dense embeddings created by the product between the original one-hot encoded sequence matrices and a look-up matrix (embedding weight) with a tunable hyperparameter for a dimension m. Additional concatenated features to the post BiLSTM representation includes melting temperature, GC content, secondary structure parameters, etc

DeepHF’s primary predictive model is based on a RNN with bidirectional long short-term memory (BiLSTM), which is selected to explicitly model the sequential and context-dependent nature of gRNA sequences. This model first maps one-hot-encoded nucleotides into a dense embedding space, which is then fed into the BiLSTM to propagate contextual information in both the 5$^ {^{\prime}}\to$3$^ {^{\prime}}$ and 3$^ {^{\prime}}\to$5$^ {^{\prime}}$ directions. The model concatenates both forward and backward directional information for a more comprehensive representation. Subsequently, known biochemical features are also merged with the LSTM outputs, which is followed by a regression process through fully connected layers for the final indel frequency predictions (figure 5).

Data preprocessing and hyperparameter tuning can be important for a successful machine-learning implementation. CRISPRpred(seq) [71] is a sequence-based sgRNA activity prediction tool, designed with conventional machine-learning mechanisms that, within constraints, can reach the same performance as DeepHF and outperforms DeepCRISPR deep learning-based approaches. This method follows a systematic approach to gradually increase the accuracy of the model in comparison with the other deep-learning-based models. General workflow starts with dataset [61, 65] preparation for a 5-fold cross validation scheme, followed by ERT for the feature extraction procedure based on the Gini score [72], resulting in a total of 1957 features for the training of DeepCRISPR dataset. This optimized dataset is trained on an SVM model, first for the purpose of hyperparameter tuning and then is retrained for the final product. Among the 4 cell types in the dataset, CRISPRpred(seq) outperforms DeepCRISPR slightly but convincingly in 3 cell types, underscoring the importance of data preprocessing and hyperparameter tuning. While its performance predictably dips on the highly atypical HEK293 cell line, a dataset frequently flagged for anomalous GC-content distributions and high off-target profiles, the authors attribute DeepCRISPR’s superior performance on this specific subset to potential data-leakage stemming from overlapping sequences during its massive unsupervised pre-training phase.

### 4.5. Addressing RNA dataset limitations

Scarcity of high-quality data has been a great challenge for many RNA-targeted machine-learning applications, including sgRNA optimization, especially when it comes to the usage of niche Cas proteins. This issue is especially apparent when applying available models on unseen cell types, indicating a lack of generalizability [73]. One of the most utilized methods in overcoming this specific issue is the usage of transfer-learning alongside high-quality, problem-specific data for fine-tuning purposes.

**Figure 6.**
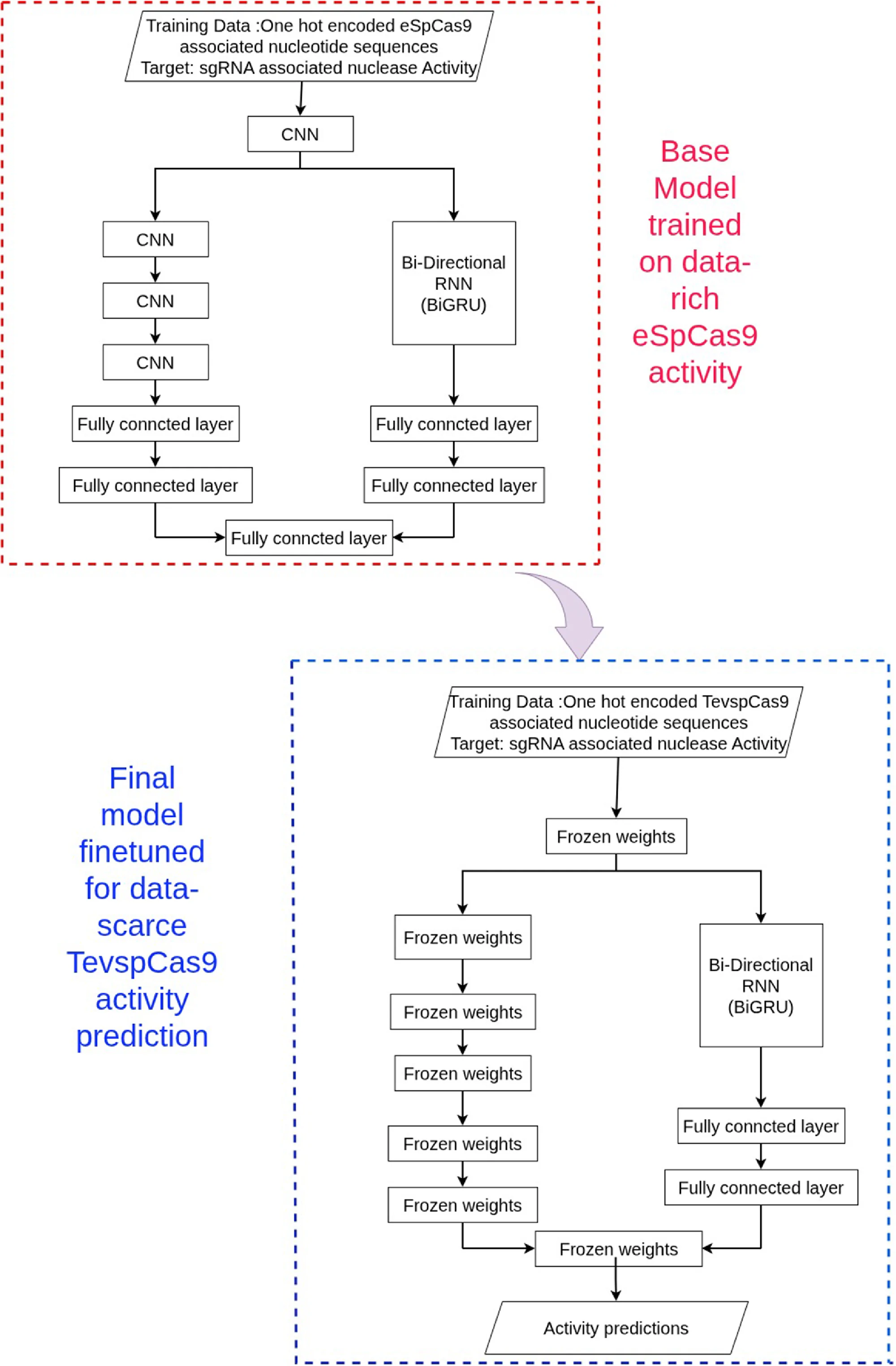
Architectures of crisprHAL base model eSpCas9 (red) and target model TevSpCas9 (blue) :Between SpCas9 and eSpCas9 trained base models, eSpCas9 outperforms the other when fine-tuned for TevSpCas9. Furthermore Freezing the weights in the common CNN head combined with the CNN only branch with a fully connected layer has been identified as the optimal combination.

A set of prominant models that follow this principle for sgRNA optimization is crisprHAL model and its subsequent iterations [74, 75]. Unlike models that rely entirely on eukaryotic frameworks or local autoencoders, crisprHAL leverages a cross-domain supervised transfer-learning architecture. Specifically, the model employs SpCas9 and eSpCas9 datasets [76] for the training of two base models of transfer-learning and TevSpCas9 dual nuclease [77] activity data created from a two-plasmid positive selection system. To compensate for the varied activity dynamics among the different Cas enzymes, rather than using raw activity data, standardized log-ratio-based relative difference is used as the target. The model architecture starts with a common CNN layer and then follows a dual branched pipeline where one branch is a four-layer CNN and the other is a combined CNN and Bi-directional gated recurrent unit (BiGRU) [78] RNN (figure 6).

This architecture is suited for sgRNA understanding because it is a problem requiring both local and global attention. CNNs are highly effective in local pattern detections, whereas BiGRU is effective in capturing long-range dependencies such as order and directionality. For the optimization of the final model, it is discovered that freezing the weights of the common CNN layer and the CNN-only branch gives the best predictions. Of the two base models, the eSpCas9-trained model outperforms the spCas9-trained model on TevSpCas9 (a dataset of just 279 entries: n=279) activity predictions.

In an attempt to examine the generalization of the model for the extension to other cell families, the model is implemented to predict sgRNA activity for targeting C. rodentium chromosome [79] (n=31796) and a pooled library of sgRNAs targeting a 2 kb katG fragment cloned into pTox (n=296) leading to correlations of >0.6 with the experimental results.

Novelty of this model lies in its transfer-learning-based framework, which enables effective generalization across diverse gene families and supports accurate activity prediction even in regimes where only relatively very small, high-quality datasets are available.

One of the most notable methodological transitions in machine-learning approaches for gRNA optimization problem is the shift from a classification task to a regression task. Rather than hard-constraining targets such as cleavage efficiency and activity to binary on-off cases, the majority of the models preserve the natural continuity of the data. A clear difference can also be observed in feature preprocessing, where early models (Azimuth) prefer the selection, manipulation, and engineering of features such as GC fraction and melting temperature, whereas newer methods (DeepCRISPR, crisprHAL) predominantly employ representation learning to automatically extract informative features directly from sequence and contextual data. This transition reflects a broader movement away from expert-designed feature engineering toward data-driven latent representations as datasets have grown in both size and complexity. Although this is true in many cases, some studies focus on determinant identification and therefore require classical machine-learning approaches/preprocessing such as RF and XGBoost, which provide explicit, interpretable feature-importance metrics, as opposed to the implicit, hidden behavior of deep-learning architectures (kCRISPR, DeepHF, CRISPRpred(seq)). The necessity of this interpretability is especially apparent in models that incorporate specific biological features into the training process, such as kinetics, chromatin, or local environmental context, as opposed to models that primarily focus on sequence-derived information. Ultimately, we find that transfer learning has emerged as a key strategy for overcoming data scarcity in specialized systems, such as eCas9, while genome language models increasingly provide a unified foundation for encoding sequence context across a broad range of CRISPR applications.

## 5. Machine learning-based optimization extended to alternate CRISPR modalities

### 5.1. Base editing incorporates optimization of key features

Engineering and optimization of BEs for improved efficiency is an active area of research. To address the challenge of mitigating the unpredictable downstream outcomes induced by double-stranded breaks, it is important for the optimization of the CRISPR system as a whole. BE-hive [80] is a model to explore the important factors that determine base-editing outcomes. This machine learning model is two-pronged and targets two important aspects of base editing, namely editing efficiency and bystander editing probabilities. The editing-efficiency model follows a classical supervised-learning pipeline while building an enhanced contextual representation by featuring not only the target-editable base but also neighboring bases to account for the sequence dependence often observed in deaminases. Coupled with multiple features, the model leverages LR and gradient-boosted regression trees (GBRT) methods to train an efficiency-prediction model. By design, the model predicts *Z*-scores for efficiency instead of raw efficiency scores, addressing possible skewing artifacts induced by population bias, and recovers raw efficiencies that are correlated with the experimental results across different BEs (R>0.5 for LR and R>0.6 for GBRT). Moreover, the model determines that descriptors such as sgRNA melting temperature and G/C fraction are critical for efficiency prediction. In contrast, the bystander-editing model captures the joint probability distribution over base-editing windows that include non-target nucleotides near a target nucleotide. Notably, The model recovers the underlying interaction information by predicting an editing pattern. Relying on the autoregressive paradigm used in language models, the bystander-editing model uses a Bayes-rule-based Deep Conditional Autoregressive neural network (DCA) [81]. This model encodes each nucleotide, including its locus, into a latent space before passing it through a decoder that iteratively generates probabilistic information describing base-editing outcomes conditioned on previously generated outputs.

DeepBE [82] is a model that adopts an end-to-end deep-learning architecture to evaluate the viability of BEs through deep-learning-based efficiency and editing-outcome predictions. It models optimal Cas9 nickase selection (including the PI domain responsible for PAM recognition) by considering two main determinants in base editing: PAM dependence (as discussed in section 2) and editor-specific behavior. DeepCas9Variant, a precursor to DeepBE, employs experimentally characterized PAM affinities of engineered Cas9 variants to compute the suitability of a given nuclease for a specific target sequence. Unlike the PAM prediction and engineering approaches discussed in section 2 (Protein2PAM), this model does not directly modify or speculate on PAM specificities but instead maps a given target sequence to an optimal Cas9 variant. In the main DeepBE architecture, the raw target sequence (including flanking context) is processed through CNN layers to extract local latent sequence features. These latent representations are subsequently concatenated with the target-specific PAM affinity vectors generated by DeepCas9Variant with explicit feature embeddings representing the selected editor variant. Based on this unified, extended input space, DeepBE trains and evaluates 63 nickase-deaminase combinations through a multi-task DNN and predicts how specific editor compositions, local PAM-recognition dynamics, and deaminase kinetics act together to determine final editing outcomes.

### 5.2. PegRNA optimization improves Prime editing applications

The more sophisticated and flexible prime-editing approaches have also been explored for machine-learning-aided optimization. PRIDICT [83] is a model trained using sequence features to predict editing efficiencies and the rates of unintended editing in prime editors. Experimental analyses identified that features such as consecutive *T* bases can detrimentally affect editing efficiency. Furthermore, features such as PBSs with higher melting temperatures, longer RTT overhangs, less insertions, higher local GC fraction were found to be favorable for achieving a higher editing efficiency. The PRIDICT model consists of three encoders: two bidirectional attention-based gated RNNs that project the target and edited sequences into a latent space, and one feed-forward network that learns a representation of the experimentally determined pegRNA features. A decoder is then used to map the final latent vectors to a probability distribution over intended editing, unintended editing, and no editing. This model achieves Spearman’s correlations of approximately ≈0.80 for both editing efficiency and unintended editing rates. Subsequent versions, PRIDICT2.0 and ePRIDICT [84, 85], expand the model to treat broader editing instances (insertions<5 bp and indels<15 bp) and account for local chromatin context, respectively. Rather than introducing a fundamentally different modeling paradigm, PRIDICT2.0 adopts the PRIDICT architecture in a transfer-learning framework with a more diverse training corpus, resulting in multi-task training across cell types. PRIDICT2.0 outperforms its predecessor and demonstrates the importance of an appropriate data distribution for the training task. However, neither of these models accounts for the local chromatin context, which can be an important determinant of the accessibility of a genomic locus for a given pegRNA. To address this limitation, a parallel model is trained to predict the influence of chromatin context based on linear and tree-based methods. The combination of these two models outperform the PRIDICT2.0 alone by approximately 20% in correlation between predicted and experimentally measured prime-editing efficiencies prediction, highlighting the importance of contextual awareness of the system in machine-learning approaches for CRISPR optimization.

The complexity of base-editing goes beyond cleavage-efficiency and hence the optimization of base-editing requires greater attention in feature engineering. This demands automated feature-extraction techniques (BEhive) and subsequent latent-space representations (DeepBE). To capture greater context awareness, more sophisticated prime-editing models (PRIDICT) utilize encoders to account for the influence of specific environmental information. Furthermore, the machine-learning tasks evolve from simple regression problems to multi-task deep-learning models (PRIDICT2.0) and ultimately to multimodal contextual learning frameworks (ePRIDICT). While the feature sets become increasingly rich with biological context, such as editor identity and chromatin information, the optimization objectives also evolve from simple activity scores to editing-outcome distributions, bystander-editing probabilities, and optimal system combinations.

In table 1 we briefly summarize the reviewed models, their modalities, formulations, and key limitations from a machine-learning perspective.

**Table 1.** Summary of the machine-learning models discussed in this article.

Model	Objective	Modality	ML formulation	Input → output	Major limitation
Protein2PAM	PAM prediction	Type I/II/V DNA-targeting systems	Transfer-learning (pLM) / representation learning + supervised regression (MLP)	Cas protein sequence → PAM preference	Limited by known Cas-PAM annotations

PAMmla	PAM-engineering	SpCas9 variants	Supervised regression (fully connected NN)	mutated PI-domain variants + PAMs → cleavage activity	Restricted to sampled mutational/assay space

Casboundary	Cas Cassette identification	Class 1 and Class 2 systems	Supervised classification (ERT & DNN)	Genomic data+HMM & seed matches → Cassette boundaries/Cas module annotations	Mining limited to existing genomic space and density-based boundary detection constraints

AIL-Scan	Cas protein mining	Class 1 and Class 2 systems	Transfer-learning (pLM) / representation learning + supervised classification (LightGBM & CatBoost)	Cas protein sequences → Cas type labels, candidate protein sequences	Relies heavily on evolutionary information being sufficient for Cas mining

OpenCRISPR	Cas protein generation	Natively covers type II systems	Transfer-learning (pLM) / autoregressor	Cas proteins + tracrRNAs + PAMs → Cas-family-oriented protein sequences	Functional viability of the generated candidates is not guaranteed

Azimuth	Cleavage efficiency prediction	Type II systems	Supervised regression	Target nt sequence → On-target efficiency score	Limited to classical machine-learning models + Information loss in high feature-engineering

DeepCRISPR	On- / Off-target activity prediction	Type II systems	DCDNN autoencoder-based representation learning + supervised regression (CNN)	Target nt sequence → On-target cleavage efficiency + Off-target cleavage probability	Migrating beyond 4 target cell lines can induce misalignment in input features yielding suboptimal results

kCRISPR	On- / Off-target activity prediction	Type II systems	OOB error analysis for feature ranking / Random forest regression	Target nt sequence → On- and Off-target cleavage efficiencies	Manual feature-engineering may lose some non-trivial information
DeepHF	On-target activity prediction	Natively covers Type II systems	XGBoost+SHAP analysis for feature ranking / supervised regression(BiLSTM RNN)	Target nt sequence + Filtered / Ranked features set → On-target efficiencies	Manually determined original feature set may overlook some non-intuitive but relevant features

CRISPRpred(seq)	Cleavage efficiency prediction	Type II systems	ERT+Gini analysis for feature ranking / supervised classification & regression (SVM)	Target nt sequence+Extracted features → activity classification & relative efficiency score	Usage of DeepCRISPR dataset invokes same generalization constraints

crisprHAL	Cleavage efficiency prediction	Type II systems	Supervised transfer-learning / regression (CNN / CNN + BiGRU RNN)	Target nt sequence → relative efficiency score	Due to transfer-learning happening on a supervised eukaryotic base model composition bias can occur

BE-hive	Base editing outcomes prediction	Base editing systems	supervised regression (LR/GBTR) for editing efficiency model & DCA for subsequent bystander probability prediction	Target window sequence + experimental environment features (base editor variant, gRNA melting temperature, etc) → editing efficiency & bystander editing probability	Limited to editor architectures represented in training data

DeepBE	Base editor viability prediction	Base editing systems	supervised regression (DNN)	PAM compatibility (DeepCas9Variant) + target sequence embeddings (1D-CNN) + editor features → editing efficiency & product genotypic patterns	Limited by the Cas9 space trained in the DeepCas9Variant model

PRIDICT	pegRNA optimization	Prime editing systems	Representation learning (bi-directional attention-based RNN:original and edited targets, feed forward network:pegRNA features) + supervised compositional regression (Decoder)	target sequence embeddings (1D-CNN) + editor feature embeddings (ePRIDICT extension includes chromatin context) → editing efficiency & product purity score	Since the model invokes fixed-window lengths to rightfully capture local context, though it is successful for small to large insertions/edits it does not generalize to large cases

## 6. Conclusion and perspectives

Machine-learning approaches have been increasingly adopted across computational biology and have been particularly impactful in the study and engineering of CRISPR–Cas systems, where they are used to optimize overall editing efficiency, mitigate off-target effects and other system-specific corrections. The models discussed in this review focus on improving specific steps of CRISPR-based genome editing through either predictive or generative strategies. Collectively, these artificial intelligence-driven methods have demonstrated strong translational success, enabling experimental applications with high accuracy and reliability.

**PAM optimization:** Models addressing PAM recognition use machine-learning-based approaches, including LLMs, to search for alternative PAM sequences and data-informed expansions of the PAM space for a given protein sequence. Furthermore, studies have shown remarkable experimental success following machine-learning-based predictions, suggesting that in silico approaches are viable for PAM-specific applications.

**Protein-engineering:** Expanding the usable protein repertoire beyond canonical effectors such as Cas9 and Cas12a remains a major research focus in the CRISPR community. Protein-engineering has demonstrated substantial promise in both identifying alternative functional nucleases through scanning both genomes and meta-genomes and developing new molecular modalities to overcome the adverse side-effects of general Cas enzymatic systems, including base-editing systems. Collectively, these advances represent significant progress in broadening the scope and versatility of CRISPR-based applications.

**sgRNA design:** sgRNA design remains one of the most fundamental aspects of the CRISPR process, as it governs not only the targeting of specific genomic loci but also the efficiency and specificity of DNA binding and cleavage. Owing to the data-rich nature of this problem, numerous machine-learning models have been developed to address key challenges, including on- and off-target activity prediction, identification of the determinant sequence and structural features influencing binding and cleavage, and strategies for learning effectively from limited datasets. The majority of the computational findings were backed by favorable experimental results indicating the importance of in silico studies.

Beyond the canonical nuclease-mediated genome editing, machine-learning approaches have been increasingly applied to specialized editing modalities such as base and prime editing. Unlike conventional CRISPR optimization, these systems introduce additional determinants, including editing-window features, bystander editing, pegRNA design, and sequence preferences. The machine learning models can effectively capture these complex editing outcomes, facilitating the optimization of such systems while reducing the need for extensive experimental screening.

The progression of the models reflects the rapid evolution of machine-learning methodologies. As these approaches continue to mature, their increasing feature-rich capability is expected to further accelerate advances in CRISPR-Cas technologies. Collectively, the studies illustrate a transition from conventional classification and regression frameworks toward feature engineering, representation learning, transfer learning, and, more recently, generative modeling. A central conclusion emerging from this review is that different machine-learning paradigms naturally align with distinct CRISPR optimization objectives and, by extension, that no single machine-learning paradigm is universally optimal; rather, the underlying biological objective largely determines the appropriate computational formulation. Classification frameworks are ideal for discovery tasks, such as identifying candidate Cas proteins in genomic and metagenomic datasets or categorizing molecules based on experimentally defined activity thresholds. In contrast, regression approaches are well suited for modeling continuous biological quantities, including cleavage and editing efficiencies, and providing more quantitative descriptions of CRISPR activity. Conversely, if we were to adopt an evolutionary perspective, conventional machine-learning methods, task-specific deep-learning architectures, and pretrained protein or genomic language models offer complementary strengths and are suited to distinct data regimes. Conventional approaches, including random forests, SVMs, and gradient-boosting methods, are computationally highly efficient and often perform well on small-to-moderate datasets, given informative biological descriptors are available for the task. Nevertheless, their performance is constrained by the quality and completeness of the descriptors/features, and they may fail to capture convoluted nonlinear relationships or long-range dependencies in biological sequences that require a holistic context. In contrast, deep-learning architectures, such as CNNs, RNNs, and transformers, learn hierarchical representations directly from sequence or experimental data, reducing dependence on a priori assumptions about feature specifications and thus improving predictive performance when sufficiently large and representative labeled datasets are available. However, these advantages come at the cost of greater computational demand, reduced interpretability, and increased susceptibility to overfitting, demanding careful dataset preprocessing and model design. Protein and genomic language models extend representation learning by pretraining on large collections of unlabeled sequences, thereby incorporating broad statistical and evolutionary information before adaptation to a CRISPR-specific task. They are therefore especially valuable when labeled data are limited and may support transfer across Cas families, organisms, or optimization objectives. Nevertheless, their substantial computational requirements, dependence on the composition of the pretraining corpus, and limited capacity to distinguish sequence correlation from causal biological function remain constraints. In summary, conventional methods may be preferable for smaller, structured, and interpretation-sensitive problems, whereas task-specific deep-learning models are advantageous for large, well-annotated datasets. Pretrained language models are particularly important in data-scarce settings that require transferable representations. Hybrid strategies that combine pretrained embeddings with simpler supervised models offer useful trade-offs among predictive performance, data scarcity, and computational efficiency.

Beyond improving prediction, expanding the editable space of CRISPR systems has become an important objective in recent years, one that generative modeling addresses directly by enlarging the design space of CRISPR systems and, consequently, the targetable genomic space. This enables the progression from prediction to design, facilitating exploration of previously inaccessible regions of CRISPR sequence space and the rational development of novel proteins, editors, and functional variants.

Nevertheless, our current understanding of these systems remains incomplete, owing to their inherent complexity in the interplay between multiple molecular processes and the limited availability of intracellular contextual information.

One of the fundamental issues in machine-learning-driven CRISPR optimization is the overwhelming data bias toward type II systems. For the collective development of CRISPR technologies, more high-quality experimental data are required, especially for modalities such as RNA-targeting systems and prime editors. It is also important that these datasets extend to previously unseen cell types. Throughout the reviewed studies, we repeatedly observe that generalization across cell types remains suboptimal and inconsistent in almost all models, whereas the performance of family- or cell-type-specific models remains favorable. This raises the question of whether current machine-learning paradigms are sufficiently sophisticated to adequately encode CRISPR mechanisms when sufficient problem-specific data are available. It should also be noted that CRISPR, as a major biological system of interest, lacks standardized benchmarking datasets for rigorously comparing the performance of different models. This is especially true in the context of gRNA optimization because of the abundance of homologous guides consistently appearing in datasets, which may cause data leakage between training and test sets if not curated properly. Therefore, the volume and standardization of CRISPR datasets should be increased. Nevertheless, it can also be argued that generalization across cell types, families, and even species is constrained by the representational capacity of the chosen learning models. This is where appropriate model selection becomes particularly important. Across the reviewed studies, deep-learning models consistently outperform classical machine-learning approaches by identifying more complex patterns and capturing richer biological information. Although this is highly valuable, the limited interpretability of these approaches remains a notable drawback. Consequently, some recent models adopt classical machine-learning algorithms to identify key determinants of CRISPR mechanisms, sacrificing the ability to capture hidden biological dynamics in the process. Moreover, improvements targeting individual steps of the CRISPR workflow may compromise other components of the editing process. Nearly all of the reviewed models, including the most recent approaches, optimize a limited set of key determinants while overlooking other biological context, such as chromatin accessibility and the cellular environment. Consequently, future studies should move beyond the isolated optimization of individual CRISPR components or single performance metrics. Adopting holistic, system-level frameworks that jointly optimize multiple objectives, as demonstrated by contemporary prime-editing models, should be encouraged. Target features such as cleavage efficiency, off-target activity, toxicity, and editing-outcome distributions should be considered simultaneously whenever possible. Such integrated approaches may better capture the complex interplay among the molecular processes governing CRISPR function. The challenges discussed are not independent of each other; rather, they are closely interconnected. Without standardized, leakage-resistant benchmarks, strong performance on randomly partitioned or closely related datasets could reflect sequence similarity or dataset-specific artifacts rather than genuinely transferable CRISPR mechanisms. This results in improvements within a particular dataset, not necessarily generalizing across different organisms, cell types, or modalities, each of which represents a potentially non-negligible domain shift. This issue is further exacerbated in highly parameterized deep learning models due to a lack of physical interpretability, which may obscure whether predictions rest on biologically meaningful determinants or on irrelevant correlations induced by dataset composition. Consequently, performance on held-out data should be distinguished from prospective biological validation. Future benchmarking frameworks should incorporate homology-clustered data-splitting, explicit cross-cell-type and cross-species evaluations, standardized performance metrics, and uncertainty estimates. Most importantly, computational predictions should be tested using blind experimental validation under conditions not represented in the training data. Such a hierarchical evaluation framework would distinguish among within-distribution predictive accuracy, cross-context transferability, and genuine experimental robustness.

## Data Availability

No new data were created or analysed in this study.
